# Characterization of the Secretomes of Two Vibrios Pathogenic to Mollusks

**DOI:** 10.1371/journal.pone.0113097

**Published:** 2014-11-17

**Authors:** Stéphanie Madec, Vianney Pichereau, Annick Jacq, Mathieu Paillard, Claire Boisset, Fabienne Guérard, Christine Paillard, Jean-Louis Nicolas

**Affiliations:** 1 Laboratoire Universitaire de Biodiversité et Ecologie Microbienne (EA3882), SFR48 ScInBios, Université de Bretagne Occidentale (UBO), UEB, ESIAB, Technopôle Brest Iroise, 29280, Plouzané, France; 2 Laboratoire des Sciences de l’Environnement Marin, UMR 6539 UBO/CNRS/IRD/Ifremer, Université de Bretagne Occidentale (UBO), Institut Universitaire Européen de la Mer, Technopôle Brest Iroise, 29280, Plouzané, France; 3 Institut de Génétique et de Microbiologie, UMR8621, CNRS-Université Paris-Sud, 91405, Orsay, France; 4 Centre de Recherche sur les macromolécules végétales, CERMAV-CNRS, BP53, 38041 Grenoble, France; Cornell University, United States of America

## Abstract

*Vibrio tapetis* causes the brown ring disease in the Japanese clam *Ruditapes philippinarum* while *Vibrio aestuarianus* is associated with massive oyster mortalities. As extracellular proteins are often associated with the virulence of pathogenic bacteria, we undertook a proteomic approach to characterize the secretomes of both vibrios. The extracellular proteins (ECPs) of both species were fractionated by SEC-FPLC and *in vitro* assays were performed to measure the effects of each fraction on hemocyte cellular parameters (phagocytosis and adhesion). Fractions showing a significant effect were subjected to SDS-PAGE, and proteins were identified by nano LC-MS/MS. 45 proteins were identified for *V. aestuarianus* and 87 for *V. tapetis*. Most of them belonged to outer membrane or were periplasmic, including porins or adhesins that were already described as virulence factors in other bacterial species. Others were transporter components, flagella proteins, or proteins of unknown function (14 and 15 respectively). Interestingly, for *V. aestuarianus*, we noted the secretion of 3 extracellular enzymes including the Vam metalloprotease and two other enzymes (one putative lipase and one protease). For *V. tapetis*, we identified five extracellular enymes, i.e. two different endochitinases, one protease, one lipase and an adhesin. A comparison of both secretomes also showed that only the putative extracellular lipase was common to both secretomes, underscoring the difference in pathogenicity mechanisms between these two species. Overall, these results characterize for the first time the secretomes of these two marine pathogenic vibrios and constitute a useful working basis to further analyze the contribution of specific proteins in the virulence mechanisms of these species.

## Introduction

Vibrios have frequently been associated with bivalve mortalities, essentially at the larval stage but also in adults [1]–[4]. Since 1987, several mortality events have been reported in clams (*Ruditapes philippinarum)* in different sites of the French coastline. Before death, clams go back to the sediment surface and display a brown deposit on the inner surface of the valves, between the pallial line and the edge of the shell [5]. This disease, named the Brown Ring Disease (BRD), was also described in Spain and Portugal, and affects both reared and wild clams. Bacteriological studies led to the identification of a new bacterial species, *Vibrio tapetis*, capable of reproducing the BRD in healthy animals [6].

In France, shellfish production is a well-established industry mainly relying on the commercial farming of the Pacific oyster (*Crassostrea gigas*). Annual mass summer mortalities of C. *gigas* have been reported since 1980 on the French coast. Several studies have demonstrated that these mortality outbreaks resulted from complex interactions between the physiological and/or genetic status of the oysters, environmental factors, and one or more infectious agents, among which the herpes virus, OsHV1 [7], and *Vibrio* sp. [8]. Analyses of both moribund and healthy oyster hemolymph revealed that *Vibrio aestuarianus* was the most frequently disease-associated species [2] until 2008. Since then, a more virulent pathogenic herpes virus OsHV1, genotype microvar, emerged, reducing the occurrence of *V. aestuarianus* while *V. splendidus* strains are still frequently isolated [9].

The observed variable virulence of the isolates could be linked to the varying toxicity of the bacterial extracellular products (ECPs), allowing bacteria to escape the host immune defenses. In a previous study, the ECPs of the pathogenic strain *V. aestuarianus* 01/32 were shown to cause lethality in *C. gigas*, as well as morphological changes and immunosuppression in oyster hemocytes [10]. Further biochemical and genetic approaches evidenced the major role of the *Vam* extracellular metalloprotease in the toxicity of *V. aestuarianus* ECPs and in the impairment of oyster hemocyte functions [11].

As for *V. tapetis*, the causative agent of BRD in adult clams, the pathogenicity process is not well established yet. As in the case of *V. aestuarianus*, *V. tapetis* isolates display variable levels of virulence [12]. This vibrio is known to decrease both hemocytes viability and phagocytic activities in *R. philippinarum* [13]. *In vitro* experiments showed adhesion capability of *V. tapetis* to the clam hemocytes and mantle cells [14], and its cytotoxic effects after phagocytosis resulted in cell rounding with loss of filipods [12].

It is recognized that the success of each step of the bacterial virulence process depends on the orchestrated activity of several specialized bacterial factors. In vibrios, such virulence factors have been more identified in human pathogens such as *V. cholerae*, *V. parahaemolyticus* and *V. vulnificus* [15]–[17] but also in *V. anguillarum*, *V. harveyi* and other fish, crustacean and mollusk pathogens [18]. Currently, the only virulence factor characterized in *V. aestuarianus* is the secreted zinc metalloprotease, Vam, a member of the thermolysin family [11]. No similar virulence factor has been described to date in *V. tapetis*, but a metalloprotease (Vsm), a homolog of Vam, was also identified as a major determinant of the toxicity of *V. splendidus* ECPs [19]. All this reinforced our objective to search for other secreted proteins potentially involved in the virulence of these two marine vibrios. So far only two vibrio secretomes have been described [20], [21] and the importance of the extracellular compartment on host pathogen interaction led us to analyze more precisely the proteins of this compartment in both vibrios.

## materials and methods

### 1. Bacterial strains, growth and culture conditions

*V. aestuarianus* 07/115 was isolated from the hemolymph of an oyster collected at Aber Benoît (Brittany, France) in September 2007. It was identified by the sequencing of the 16S rRNA and *gyrB* genes and was found to be highly virulent when injected in adult oysters (Jean-Louis Nicolas, unpublished results). The *V. tapetis* CECT4600 strain was isolated in Aber Benoît (France) in Landeda (France) in 1995 from BRD diseased Manila clam (*Ruditapes philippinarum*) [22]. These strains were respectively grown in Difco marine broth 2216 (BD, Franklin Lakes, USA) and Zobell broth (HiMedia Laboratories, Mumbai, India), or on Difco marine agar and Zobell agar at 18°C.

### 2. Preparation of extracellular products (ECPs) and fractionation by Size Exclusion Chromatography in Fast Purification Liquid Chromatography (SEC-FPLC) mode

ECPs were produced by the cellophane overlay method as previously described [10]. Total ECPs of *V. aestuarianus* and *V. tapetis* culture supernatants were filtered through 0.22 µm filter membranes and concentrated on an Amicon Ultra-4 membrane with a 10,000 molecular weight cut-off (MWCO) (Millipore, Billerica, MA, USA). The total protein content was quantified using a DC protein assay (Bio-Rad, Hercules, CA, USA) with 96-well micro-plates (Nunc) in a micro-plate reader (Bio-Tek Synergy HT) and the KC4 v3 software comparing the results with a calibration curve using standard proteins (Bovine Serum Albumin) provided with the DC protein assay kit. Then, ECPs were separated on an ÄKTA*FPLC* system (GE Healthcare, Piscataway, NJ, USA) directed by the Unicorn 5.1 software. Aliquots containing 1.4 mg of total proteins dissolved in mobile phase (isocratic elution mode in PBS: 10 mM Phosphate Buffer pH 7.4, 137 mM NaCl, and 2.7 mM KCl) and filtered on a 0.22 µm membrane was injected onto a Superdex S200 HR10/30 gel filtration column from GE Healthcare (fractionation range of the column: 10–600 kDa) at a flow rate of 1 ml/min. Absorbance was monitored at 280 nm and 1 mL fractions were collected. The protein concentration of each fraction was determined and protease activity was assayed using azocasein as previously described [10]. Fractionated ECPs were conserved at −80°C until *in vitro* assays.

### 3. *In* *vitro* assays : hemocyte cellular parameters

The effects of the obtained fractions were measured on oyster or clam hemocytes to assess the action of the ECPs on hemocyte adherence and phagocytosis capacities. Fractions showing inhibitory or stimulatory effects were compared to the negative control (FSSW: Filtered Sterile Sea Water). For both tests, ECPs of each bacterial species were tested at 32 µg.mL^−1^ of proteins, following previously described procedures [10]. Briefly, for phagocytosis tests, a sub-sample (150 µl) of each hemolymph pool was distributed into a 5 ml polystyrene tube (Falcon, B-D Biosciences, San Jose, CA, USA), then underwent a two fold dilution with FSSW and was maintained on ice. Each sub-sample was subsequently combined with 30 µl of a fluorescent bead (2.00 µm in diameter, Fluoresbrite calibration grade, Polysciences, USA) working suspension (2% of the commercial suspension in FSSW, final concentration 1×10^7^ beads.mL^−1^), and incubated at 18°C for 120 min. The cells were then analyzed on a flow cytometer (FACSCalibur, BD San Diego, USA). The results were expressed as the percentage of hemocytes containing three beads or more [10].

To estimate hemocyte adhesive capacity, the sub-samples (100 µl) of each hemolymph pool were distributed into 24-well microplates maintained on ice, as already described by Choquet *et al*. [12]. 100 µl of FSSW or ECPs was added in triplicate to each sub-sample. After three hours of incubation at 18°C, the cells were fixed by addition of 200 µl of a 6% formalin solution in FSSW. The supernatants were then transferred to cytometry tubes. The hemocyte number present in each supernatant was determined by flow cytometry. The results are expressed as average of non-adherent cells per ml i.e. an increase of the value compared to that of the negative control shows a cytotoxic effect of the tested ECPs.

### 4. Proteins electrophoresis (SDS-PAGE)

The fractions showing a significant effect *in vitro* on hemocyte phagocytosis or adherence were concentrated with Corning Spin-X UF Concentrators (Corning, Lowell, MA, USA) with a 10 kDa MWCO and applied on a Criterion precast acrylamide gradient gel 8–16% in Tris-HCl (Biorad, Hercules, CA, USA). After staining by Coomassie blue (Biosafe Coomassie, Biorad), the gel bands were cut out manually and conserved at –20°C before trypsin digestion.

### 5. Protein identification

#### 5.1. In-gel digestions and peptides recovery

Excised gel plugs were washed 3 times with water, 100 mM ammonium bicarbonate and 100% acetonitrile successively. Cysteins were reduced by a treatment with a 65 mM DTT solution for 15 minutes at 37°C followed by alkylation with 135 mM iodoacetamide at room temperature in the dark. Gel plugs were washed again with 100 mM ammonium bicarbonate/acetonitrile (1∶1), 100% acetonitrile, 100 mM ammonium bicarbonate and 100% acetonitrile successively before being dried. Gel pieces were then re-swollen in a solution of trypsin (12.5 ng/µL in 50 mM ammonium bicarbonate; Promega), and digestion was performed overnight at 37°C. The resulting peptides were then extracted from the gel by sequential incubation in the following solutions: acetonitrile (ACN)/H_2_O/trifluoroacetic acid (TFA), 70∶30∶0.1 (v/v/v), 100% ACN and ACN/H*_2_*O/TFA, 70∶30∶0.1 (v/v/v), and extracts were eventually concentrated by evaporation to a final volume of 30 µL.

#### 5.2. Mass spectrometry (MS) analysis

Peptide mixtures were separated on a nano-HPLC system (Ultimate 3000, Dionex, Jouy-en-Josas, France), with an injection volume of **22 µ**L: first, they were concentrated into a reversed-phase C18-PepMap trapping column (**5 µ**m, 300 Å**/300 µ**m i.d. x 5****mm, Dionex), and were then eluted with a 75-min gradient of ACN (from 2 to 90%) in aqueous 0.05% formic acid, at a flow rate of 250 nL/min. The nano-LC apparatus was coupled on-line with an Esquire HCT Ultra PTM Discovery mass spectrometer (Bruker Daltonik, GmbH, Bremen, Germany), equipped with a nanoflow ESI source and an ion trap analyser (ITMS). The mass spectrometer was operated in the positive ionization mode. The EsquireControl software (Bruker Daltonik, GmbH) automatically alternated MS and CID MS-MS acquisitions with the following criteria: up to seven ions per MS scan with an intensity threshold of 30,000 and a dynamic exclusion of 15****sec.

#### 5.3. Protein identification

The DataAnalysis 3.4 software (Bruker Daltonik, GmbH) was used to create the peak lists from raw data. For each acquisition, a maximum of 2,000 MS/MS spectra were detected with an intensity threshold of 100,000 and the charge state of precursor ions was automatically determined by resolved-isotope deconvolution. The proteinScape 2.0 software (Bruker Daltonik, GmbH) was used to submit the MS/MS data to the genomic *V. aestuarianus* 02/041 database (3693 CDS sequences; 1125373 residues, unpublished results), the only *V. aestuarianus* sequences available at that time. Peptide sequences were found to be 100% identical to the identified proteins in the database. Similarly, the MS/MS data for V. *tapetis* were submitted to the *V. tapetis* CECT4600 database (5498 sequences; 1633991 residues, unpublished). Submission to randomized versions of these databases (decoy) was used to determine the false positive rate (FPR), defined as the number of validated decoy hits/(number of validated target hits + number of decoy hits)*100, using the Mascot algorithm (Mascot server v2.2.07; http://www.matrixscience.com). Trypsin was selected as the cleaving enzyme with one allowed missed cleavage. In addition, carbamidomethylation of cysteins was set as fixed modifications and methionine oxidation were considered as variable modifications. The mass tolerance for parent and fragment ions was set to 0.6 and 0.5 Da, respectively. Peptide identifications were accepted if the individual ion Mascot scores were above 25 or the identity threshold (the ion score is −10*log(P), where P is the probability that the observed match is a random event, p-value<0.05). In case of ambiguous assignments (one compound fitting more than one peptide), the peptide sequence with the highest score was retained. The compilation of peptides identified to proteins was performed with the ProteinExtractor algorithm [23], so that every protein reported was identified by at least one peptide with a significant ion Mascot score (above the identity threshold) that could not be mapped to a higher-ranking protein already in the result list. This means that the final protein lists contain only those proteins and protein variants that could be distinguished directly by MS/MS. For every protein reported in the identification lists, a combined protein score (metascore) was calculated from the peptide scores with the ProteinExtractor algorithm. Finally, protein identifications were accepted if the False Positive Rate of the search was lower than 1%.

#### 5.4. Bioinformatics

For each result of proteomic identification, we used various softwares and algorithms to determine i/a score of identification; this score was given by the MASCOT software, ii/the presence or not of a signal peptide and the predicted position of the cleavage site; the algorithm SignalP 3.0 (probability>0.93) was used except in the case of TolC for which SignalP 4.01 was used instead (http://www.cbs.dtu.dk/services/SignalP/) and iii/the subcellular localization using PsortB and Psort Gram negative bacteria (http://psort.hgc.jp/form.html); in case of ambiguity (score above threshold for two locations), the highest score was chosen. Lipoproteins and their localisation (outer membrane associated versus inner membrane associated) were predicted using LipoP1.0 (http://www.cbs.dtu.dk/services/LipoP/). In general, lipoproteins are periplasmic but anchored to one or the other membrane by their acyl moiety (indicated by P/OM for instance). In most cases, they were associated with the OM. In some cases, they could be associated with the OM and facing outward.

## Results and Discussion

### 1. Preparation and fractionation of *V. aestuarianus* 07/115 and *V. tapetis* CECT4600 ECPs

The proteins from the extracellular compartment are of particular interest for functional investigation of bacterial pathogen virulence, because they come into direct contact with host tissues and are often effectors of pathogenicity. Several lines of evidence highlight an important role of ECPs in the virulence of pathogenic vibrios. For example, a previous study on *V. aestuarianus* 01/032 showed that its ECPs displayed immunosuppressive activities on oyster hemocyte functions [10]. Similar effects were described in *V. tapetis,* in which ECPs were shown to significantly decrease adhesive- [12] and phagocytic- [13] activities of clam hemocytes. However, although the biological activity of *V. aestuarianus* ECPs has been associated with the secretion of the zinc metalloprotease, Vam [11], few studies have been carried out to date in *V. tapetis* and nothing is known about the molecular components responsible for the biological activity of the *V. tapetis* ECPs.

The extraction of secreted proteins was performed under conditions known to induce virulence [11], [12]. ECPs were fractionated, their biological activity against hemocytes was assayed, and their protein contents were analyzed, as described in materials and methods.

**Figure 1:**
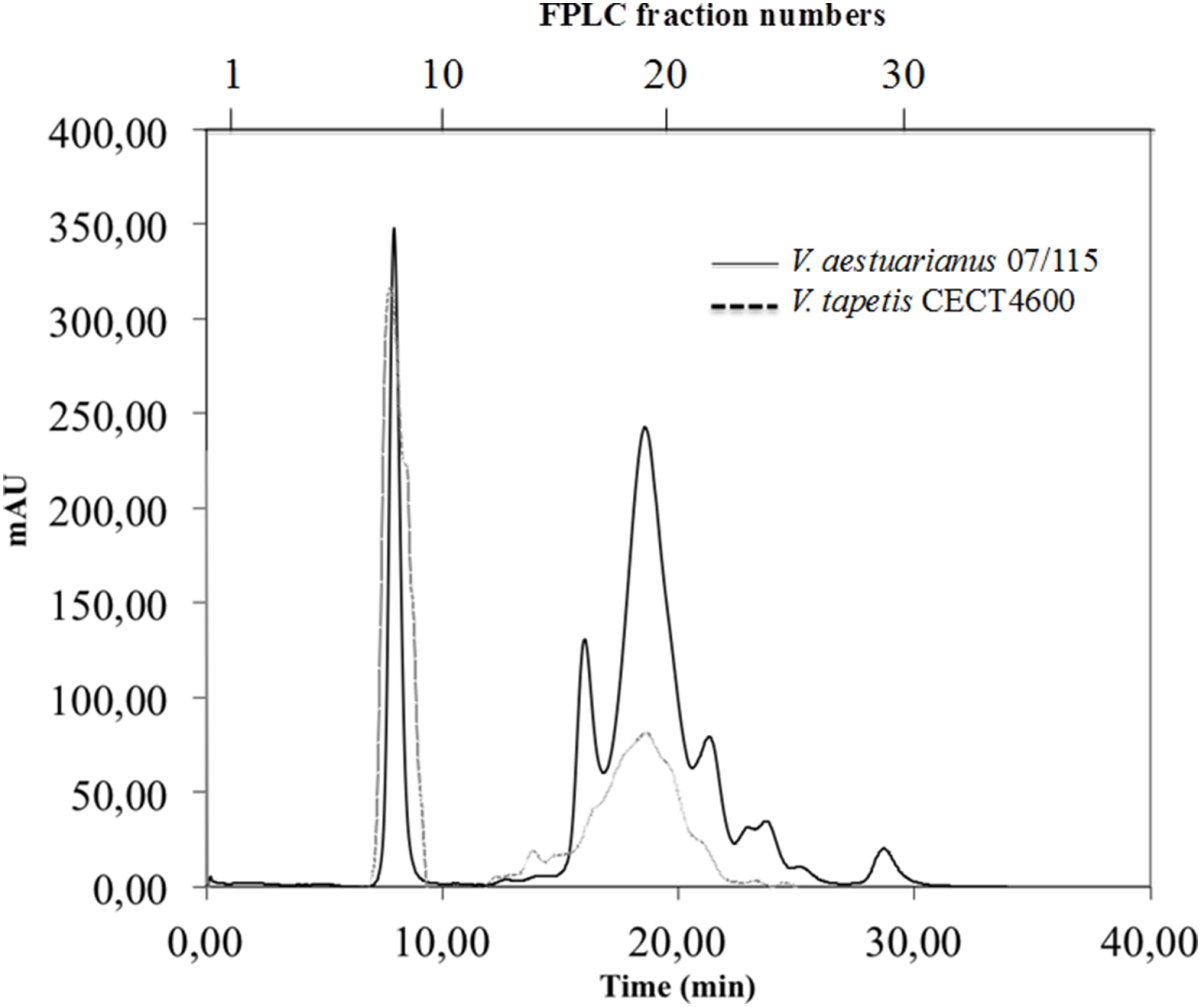
UV spectrum of total ECPs of *V. aestuarianus* and *V. tapetis* on a Superdex S200 10/30 column. Eluted fractions were collected with a flow of 1 mL/min. Fractions are numbered according to their elution time (top and bottom X-axes, respectively). Gel filtration profile was expressed in milliabsorbance units (mAU).

In the case of *V. aestuarianus*, fractionation of total ECPs gave four major peaks (Fig. 1). A first symmetrical peak eluted in the void volume of the column, suggesting that it was composed of a mixture of protein aggregates or complexes larger than 600 kDa. Three poorly resolved additional peaks eluted at 16, 18 and 22 minutes, respectively. The elution diagram obtained with *V. tapetis* ECPs comprised a first peak also eluting in the void volume, and a second broad peak, lower in absorbance than the three peaks of *V. aestuarianus,* but exactly superimposed. The fractions were recovered every minute and numbered according to the elution time. Determination of fraction protein contents allowed us to select a set of fractions (8, 9, 16 to 23 for *V. aestuarianus* ECPs and 8, 9, 14 to 21 for *V. tapetis* ECPs) showing a minimal concentration of 0,3 mg/ml of protein, to carry out further analyses.

### 2. Effects of *V. aestuarianus* and *V. tapetis* ECPs on phagocytosis and adherence activities of oyster and clam hemocytes respectively

**Figure 2:**
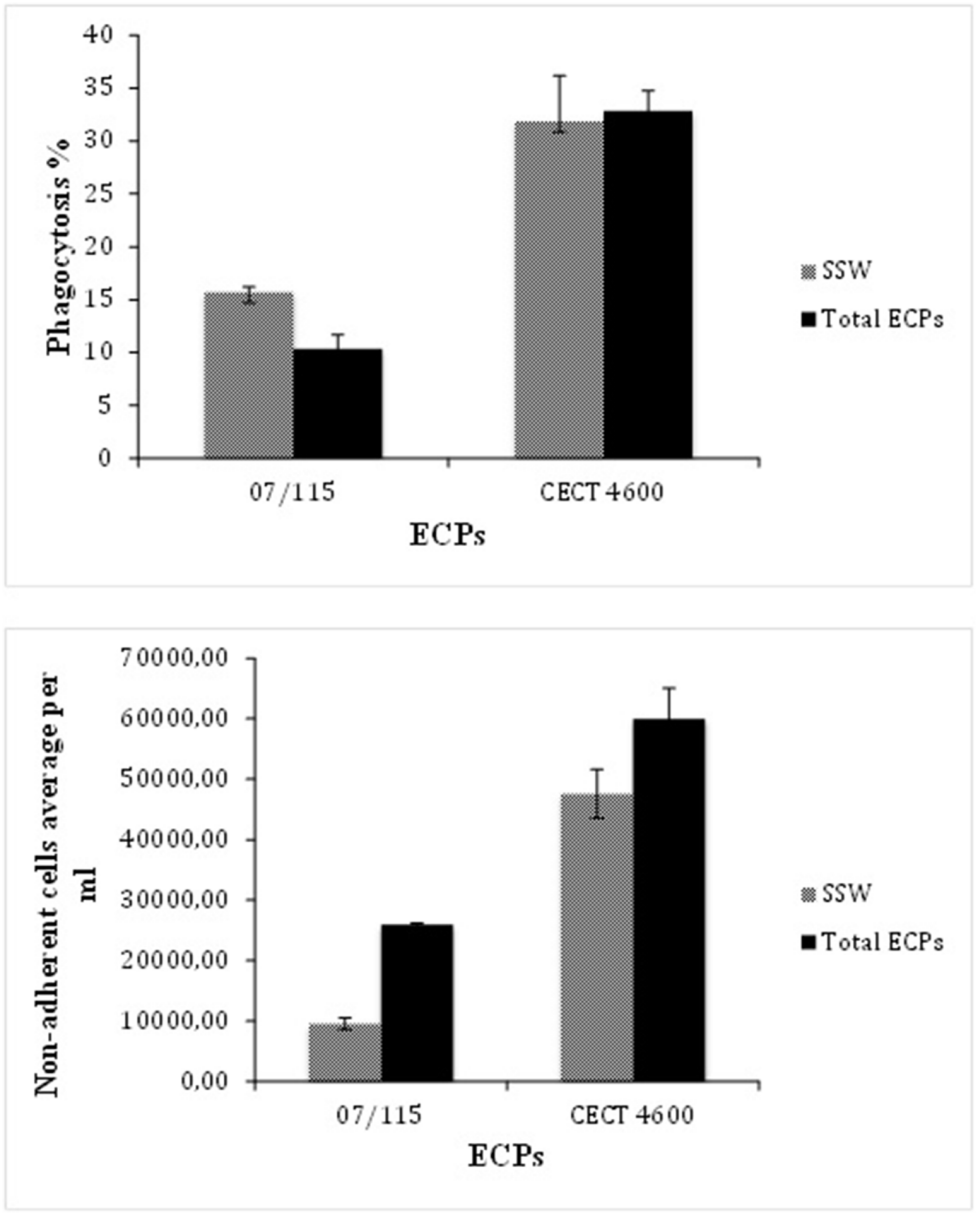
Effect of *V. aestuarianus* 07/115 (right bars) and *V. tapetis* CECT 4600 (left bars) total ECPs on oyster and clam, respectively, hemocyte phagocytosis capability (top panel) and hemocyte adhesion (bottom panel). Tests were carried out in triplicates as described in M&M and the error bars correspond to SD. Incubation of hemocytes with sterile sea water (SSW) was used as a negative control.

We first assayed the activities of unfractionated ECPs. In both cases, the biological parameters assayed were hemocyte adhesion and phagocytosis. We found that *V. aestuarianus* ECPs induced a decrease of phagocytosis and adherence properties of oyster hemocytes as shown in Fig. 2. This result is in keeping with previous results obtained by Labreuche et al. [10]. Similarly, *V. tapetis* ECPs triggered a decrease of hemocytes adherence as previously described [12]. However, *V. tapetis* total ECPs did not impact the phagocytic ability of clam hemocytes, contrary to what was found by Allam and Ford [13] who previously described a decrease in phagocytosis after treatment by bacterial supernatants obtained from liquid cultures. This discrepancy may be due to the different conditions used to prepare the ECPs (liquid culture versus cellophane overlay on plate).

**Figure 3:**
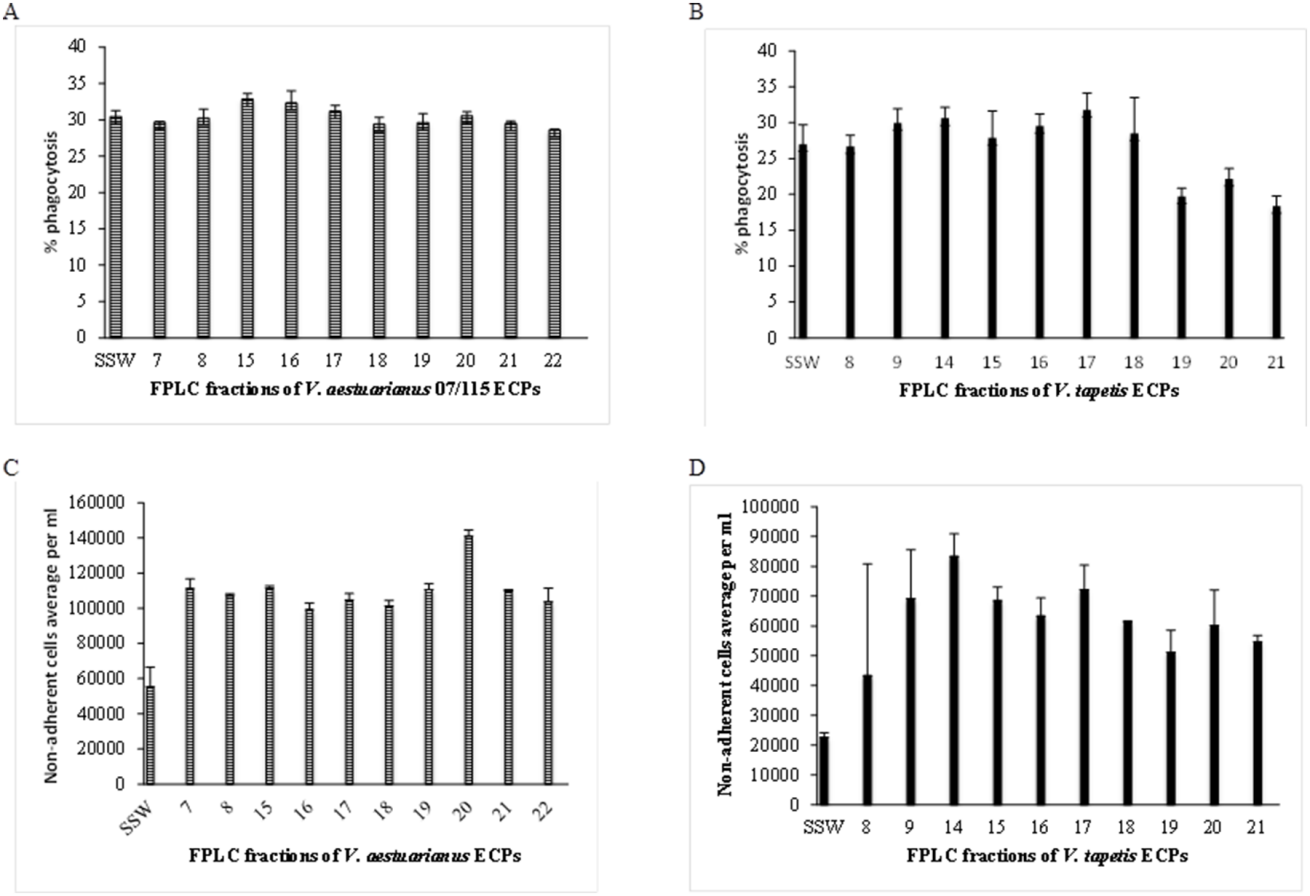
Effects of *V. aestuarianus* (panels A and C) and *V. tapetis* (panels B and D) fractionated ECPs on oyster (A and C) and clam (B and D) hemocyte phagocytosis capacity (A and B) and adhesion properties (C and D). Tests were carried out in triplicates as described in materials and methods and the error bars correspond to SD.

The different FPLC fractions of ECPs previously obtained were then similarly tested for biological activity towards oysters (*V. aestuarianus)*- and clam *(V. tapetis)*- hemocytes. The results presented in Fig. 3 showed that all the assayed fractions obtained from *V. aestuarianus* decreased the adhesive capacities of oyster hemocytes, with an increase of non-adherent hemocytes ranging from a factor 1.8 (fraction 16) to 2.5 (fraction 20). The only extracellular virulence factor described to date for *V. aestuarianus* is the Vam metalloprotease, which causes aggregation and the loss of pseudopods of oyster hemocytes [11]. Only the fractions 16 and 17 contained an azocaseinase activity (data not shown), suggesting that Vam is not responsible for this loss of adhesion and that *V. aestuarianus* 07/115 extracellular products, in particular in fraction 20, contain additional factors playing a role in adherence decreasing.

However, although total *V. aestuarianus* ECPs decreased the phagocytic activity of hemocytes (Fig. 2), none of the *V. aestuarianus* ECP fractions affected the oyster hemocyte phagocytic activity (Fig. 3A). This result suggests that phagocytosis inhibition by ECPs may involve the joined activity of several factors that have been eluted in separate fractions.

In the case of *V. tapetis* ECPs (Fig. 3B), a reduction in clam hemocyte phagocytosis capacity was recovered in fractions 19–20–21, in accordance with previously published results [13]. This suggests that an inhibitor of this activity is present in the total *V. tapetis* ECPs, which was separated during fractionation. As in the case of *V. aestuarianus*, all the recovered fractions displayed an effect on hemocyte adhesion, but with more variations amongst fractions. For example, fraction 14 triggered in excess of a 4-fold increase in non-adherent hemocytes whereas fraction 8 had only a 2-fold effect (Fig. 3D).

In summary, our results indicate that both *V. tapetis* and *V. aestuarianus* ECPs have major effects on hemocyte properties including loss of adherence and inhibition of phagocytosis, especially in the case of *V. tapetis*. In the case of *V. aestuarianus*, inhibition of adhesion is independent of Vam, and is maximal in fraction 20. In the case of *V. tapetis*, we could partly separate adhesion inhibition activity (maximal in fraction 14) and phagocytosis inhibition activity (maximal in fractions 19–21). In contrast, the observed phagocytosis inhibition in *V. aestuarianus* was lost upon fractionation, suggesting that it requires several factors acting in a complementary way while with *V. tapetis*, it was detected only after fractionation, suggesting the presence of an inhibitor in total ECPs.

### 3. Proteomic analysis of the two secretomes

**Figure 4:**
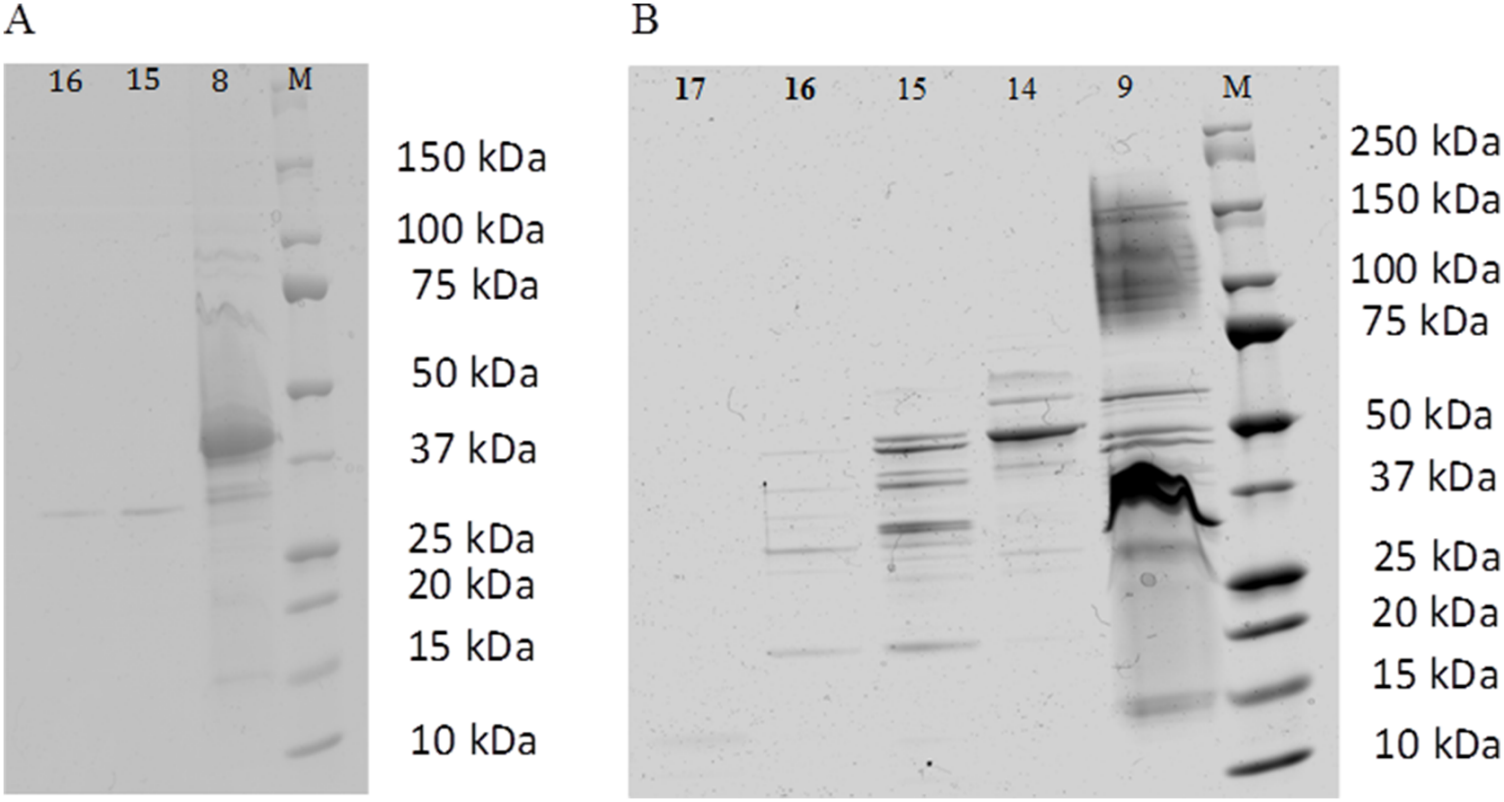
SDS-PAGE of different FPLC fractions showing a significative effect on hemocyte adherence and/or phagocytosis properties: fractions 8A, 15A, 16A for *V. aestuarianus* (A) and 9T, 14T, 15T, 16T, 17T for *V. tapetis* (B).

Fractions combining both a significant effect *in vitro* on hemocytes, and sufficient protein amounts were further characterized by proteomic analysis. Accordingly, fractions 8 and 16 to 23 for *V. aestuarianus* and 9, 14 to 17 and 19 to 21 for *V. tapetis* were subjected to SDS-PAGE for further protein identification by nano LC-MS/MS. Several fractions (18 to 22 for *V. aestuarianus* and 19, 20, 21 for *V. tapetis*) did not show any band after Coomassie Blue staining, but were directly trypsinolyzed and analyzed by liquid chromatography tandem mass spectrometry (LC-MS/MS) starting from a total protein content of 5 µg. As shown in Fig. 4, active fractions from both bacteria contained multiple proteins. In order to identify them, 16 and 43 distinct bands were excised for *V. aestuarianus* and *V. tapetis*, respectively, and analyzed by mass spectrometry, as described in materials and methods. Using the known proteome from both species, our proteomic analysis of *V. aestuarianus* and *V. tapetis* secretomes led to the unambiguous identification of 45 and 87 proteins, respectively (Tables 1 and 2). Only five proteins in the ECPs of *V. tapetis* and none in the *V. aestuarianus* secretome were predicted to be cytoplasmic, emphasizing the quality of our protocol and the absence of cell lysis.

**Table 1:** Proteins identified in the *V. aestuarianus* secretome.

Protein function	Genbankaccession Nr	MW (kDa)	Score*	Nr ofpeptides	coverage	FPLCfraction	Signalpeptidecleavagesite **	Predictedlocalization***
**Structural component, envelope biogenesis and quality control, stress response**								
OmpU: Outer membrane protein U	KM588597	37	1682	20	68	8	21–22	OM
Lpp: Major outer membrane lipoprotein	KM588630	9	335	4	62	8	22–23	P/OM
OmpA: Outer membrane protein A	KM588598	35	327	5	22	8	21–22	OM
BamA/YaeT: Outer membrane protein assembly factor	KM588599	91	897	17	23	8	20–21	OM
Tsp: carboxy-terminal protease for penicillin-binding protein 3	KM588600	75	198	5	8	8	23–24	P
Peptidoglycan-associated outer membrane lipoprotein Pal	KM588601	19	100	2	15	8	25–26	P/OM
Periplasmic component of the Tol-Pal system, YbgF-like	KM588602	29	453	6	25	8	23–24	P
Thiol-disulfide isomerase DsbA	KM588603	22	146	4	20	8	19–20	P
LptD: LPS-assembly protein	KM588604	52	52	2	3	8	24–25	OM
Putative lipoprotein LpoA, activator of penicillin binding protein 1A	KM588605	67	226	5	9	8	26–27	P/OM
Putative penicillin-binding protein activator LpoB-like, outer-membrane lipoprotein	KM588606	22	894	12	71	8	25–26	P/OM
NlpI lipoprotein	KM588607	37	334	7	19	8	37–38	P/OM
**Energetic metabolism**								
TorA: trimethylamine-N-oxide reductase (TMAO reductase)	KM588608	92	119	3	4	8	33–34	P
NapA: nitrate reductase, periplasmic, large subunit	KM588631	93	1012	19	24	8	29–30	P
**Transporter components**								
LamB: Maltoporin	KM588632	43	800	12	43	8	22–23	OM
Putative outer membrane porin; locus of qsr prophage	KM588610	35	117	3	12	8	19–20	OM
HisJ : ABC type histidine transporter periplasmic histidine binding protein	KM588611	28	1169	19	72	8	21–22	P
AapJ : ABC type L Aminoacid transporter subunit; periplasmic-binding	KM588611	37	720	13	49	8	25–26	P
PtsS1: ABC type phosphate transporter, periplasmic phosphate binding protein	KM588633	29	613	11	42	8	22–23	P
TRAP-type uncharacterized transport system, periplasmic component	KM588612	34	524	12	32	8	24–25	P
MglB: methyl-galactoside ABC transporter periplasmic-binding protein	KM588613	35	460	7	21	8	22–23	P
Putative ABC type dipeptide transporter, periplasmic peptide-binding protein	KM588637	57	266	6	11	8	23–24	P
Putative ABC type tungstate transport system, substrate binding protein	KM588614	30	78	2	8	8	23–24	P
Pts2: ABC type phosphate transport system, periplasmic component	KM588635	30	68	2	7	8	21–22	P
TolC: outer membrane efflux channel of type I secretion system	KM588615	48	64	2	5	8	22–23	OM
FadL2: Long-chain fatty acid outer membrane porin; bacteriophage T2 receptor	KM588616	46	225	4	15	8	25–26	OM
Putative YceI-like protein: lipid/polyisoprenoid-binding protein	KM588636	20	89	2	8	8	22–23	P
**Motility/Flagella**								
FliD: Flagellar hook-associated protein 2	KM588617	71	123	3	6	8	-	E
**Extracellular proteins**								
Vam: secreted Zinc Metalloprotease Vam	KM588637	66	1011	14	27	16, 17	25–26	E
Putative extracellular triacylglycerol lipase	KM588638	83	631	12	16	8	20–21	E
Vpp: Vam protease processing enzyme	KM588639	101	422	6	8	8	23–24	E
**Unknown function**								
**Lipoproteins**								
Putative outer membrane lipoprotein	KM588618	21	469	6	39	8	17–18	P/OM
Maltose operon periplasmic protein (MalM), outer membrane associated lipoprotein	KM588640	71	139	4	12	8	24–25	P/OM
Conserved lipoprotein of unknown function	KM588619	14	193	4	33	8	21–22	P/OM
Putative outer membrane lipoprotein	KM588620	20	51	1	7	8	27–28	P/OM
Conserved lipoprotein of unknown function	KM588621	12	155	3	27	8	22–23	P/OM
Conserved lipoprotein of unknown function	KM588622	14	78	1	15	8	21–22	P/OM
Putative outer membrane lipoprotein	KM588623	28	190	3	18	8	20–21	P/OM
Conserved outer membrane lipoprotein of unknown function	KM588641	21	362	9	62	8	17–18	P/OM
**Others**								
Putative outer membrane protein	KM588624	17	110	2	13	8	19–20	OM
Outer membrane proteins	KM588625	84	110	4	5	8	26–27	OM
Conserved outer membrane of unknown function	KM588626	25	83	1	6	8	23–24	OM
Conserved exported protein of unknown function	KM588627	29	356	7	41	8	18–19	P
Conserved outer membrane protein of unknown function	KM588628	35	137	2	7	8	20–21	OM
Conserved outer membrane protein of unknown function	KM588629	20	63	2	12	8	19–20	OM

* score given by the MASCOT software, ** as given by the algorithm SignalP 3.0 and some lipoproteins where putative SignalPeptidaseII cleavage sites were detected by LipoP 1.0, *** algorithm used for subcellular localization: PsortB and Psort Gram negative bacteria. Lipoproteins and their localisation were predicted using LipoP1.0 (see details in materials and methods, bioinformatics). (OM: outer membrane, IM: inner membrane, P: periplasmic, E: extracellular).

**Table 2:** Proteins identified in the *V. tapetis* secretome.

Protein function	Genbankaccession Nr	MW(kDa)	Score*	Nr ofpeptides	coverage	FPLCfraction	Signalpeptidecleavage site**	Predictedlocalization***
**Structural components, envelope biogenesis and quality control, stress response**								
OmpH: Outer membrane porin H	KM596581	35	2838	33	71	16	19–20	OM
OmpU: Outer membrane protein U	KM596582	37	2318	24	77	16	21–22	OM
OmpV: Outer membrane protein V	KM596645	28	303	6	29	16	23–24	OM
BamA/YaeT: Outer membrane protein assembly factor	KM596583	90	204	3	4	9	20–21	OM
OmpA: Outer membrane protein A	KM596646	35	665	9	27	9	20–21	OM
Lpp : major outer membrane lipoprotein	KM596647	10	54	1	12	9	22–23	OM
Outer membrane protein	KM596648	27	93	2	7	9	18–19	OM
TolB : periplasmic component of the Tol-Pal system	KM596584	49	787	17	34	9	-	P
Periplasmic component of the Tol-Pal system, YbgF-like	KM596585	29	104	1	11	9	23–24	P
Tsp: carboxy-terminal protease for penicillin-binding protein 3	KM596586	75	397	9	16	9	22–23	OM
SurA: peptidyl-prolyl cis-trans isomerase	KM596587	48	261	5	15	9	22–23	P
DegP serine endoprotease	KM596588	48	194	5	9	9	27–28	P
Putative lipoprotein LpoA, activator of penicillin binding protein 1A	KM596589	68	529	11	17	9	44–45	P/OM
MltC : membrane-bound lytic murein transglycosylase C	KM596590	45	76	2	6	9	-	IM/P
Putative MltA-interacting MipA	KM596591	50	141	3	10	9	33–34	OM
NlpI lipoprotein	KM596592	35	199	3	17	9	22–23	P/OM
SodB : iron superoxide dismutase	KM596593	21	303	5	32	15	-	C
SodM : superoxyde dismutase Mn/Fe	KM596649	23	161	4	17	15	-	C
LptD : LPS-assembly lipoprotein	KM596594	87	126	3	4	9	24–25	OM
HslJ: Heat-shock lipoprotein	KM596595	16	78	2	20	9	23–24	P/OM
**Energetic metabolism**								
TorA: trimethylamine N-oxide (TMAO) reductase I catalytic subunit	KM596596	92	754	12	14	14	33–34	P
TrxA : thioredoxin 1	KM596597	12	213	3	26	9	-	C
**Transporter components**								
Putative outer membrane efflux channel of type I secretion system	KM596598	48	136	3	8	9	19–20	OM
Putative outer membrane efflux channel of type I secretion system	KM596599	51	55	2	4	9	22–23	OM
TolC: outer membrane efflux channel of type I secretion system	KM596600	47	1434	21	54	9	22–23	OM
LamB: maltoporin	KM596650	46	249	5	16	9	24–25	OM
Outer membrane porin	KM596601	37	143	2	7	9	19–20	OM
Putative outer membrane porin	KM596602	40	436	11	34	9	22–23	OM
AapJ : ABC type L Aminoacid transporter subunit; periplasmic-binding	KM596603	36	1004	17	47	9	25–26	P
TRAP-type uncharacterized transport system, periplasmic component	KM596604	35	996	17	44	9	24–25	U
BtuB: Vitamin B12/cobalamin outer membrane transporter, TonB dependent	KM596605	68	747	13	23	9	22–23	OM
Putative chitoporin	KM596606	50	1046	14	39	9	23–24	OM
Putative (GlcNAc)2 periplasmic substrate-binding protein of ABC transporter	KM596607	63	667	13	23	9	27–28	P
Putative (GlcNAc)2 ABC transporter, periplasmic substrate-binding protein of ABC transporter	KM596608	63	152	4	7	9	23–24	P
Putative substrate binding protein component of oligopetpide/dipeptide ABC transporter	KM596609	66	643	14	27	9	20–21	P
Putative ABC-type oligopeptide/dipeptide transport system, substrate-binding periplasmic component	KM596651	69	151	4	7	9	20–21	P
Fad L1: Long-chain fatty acid outer membrane porin	KM596652	44	164	4	16	9	21–22	OM
FadL2: Long-chain fatty acid outer membrane porin	KM596610	43	441	6	18	9	25–26	OM
ABC-type sugar transport system, periplasmic component	KM596611	45	326	7	24	9	21–22	P
Putative outer membrane protein of unknown function	KM596653	40	326	6	15	9	21–22	OM
Putative ABC-type transport system, periplasmic substrate-binding component	KM596612	33	270	5	25	9	25–26	P
TRAP-type C4-dicarboxylate transport system, periplasmic component	KM596613	37	228	4	18	9	22–23	P
Putative TonB-dependent receptor protein	KM596654	77	91	2	3	9	22–23	OM
Spermidine/putrescine ABC transporter: substrate binding periplasmic protein PotD2	KM596614	39	61	1	4	9	24–25	P
Putative oligogalacturonate-specific porin	KM596615	26	522	10	26	9	20–21	OM
Rutative Rhs family protein, may bind carbohydrates	KM596616	259	54	1	1	14	-	IM
**Iron acquisition**								
Putative outer membrane siderophore receptor	KM596655	65	65	2	3	9	25–26	OM
FbpA : Periplasmic ferric iron-binding protein of ABC transporter	KM596617	37	302	6	18	9	21–22	P
Putative TonB-dependent vibriobactin receptor	KM596656	71	75	2	4	9	29–30	OM
Putative iron-regulated protein with peptidase M75 domain	KM596618	46	235	4	9	9	24–25	OM
**Catabolism**								
**Chitin utilization**								
GbpA: N-acetyl glucosamine (GlcNAc) binding protein A	KM596619	54	358	7	14	17	24–25	E
Chitinase	KM596657	53	472	8	20	15	19–20	U
ChiA: endochitinase A	KM596620	89	367	6	6	14	21–22	E
Endochitinase	KM596621	88	935	11	12	16	22–23	E
Putative chitinase	KM596658	70	1876	26	35	16	28–29	OM
Putative chitinase	KM596659	48	192	4	9	9	20–21	P
**Others**								
Aryl/Alkyl sulfatase	KM596660	73	251	6	12	9	19–20	P
Ggt : Gamma-glutamyltranspeptidase	KM596622	63	212	6	11	16	24–25	P
PepD : aminoacyl-histidine dipeptidase (peptidase D)	KM596623	53	162	5	10	14	-	C
CpdB: 2′:3′-cyclic-nucleotide 2′- phosphodiesterase	KM596624	72	67	2	2	9	25–26	P
UshA: bifunctional UDP-sugar hydrolase and 5′-nucleotidase, outer membrane associated lipoprotein	KM596625	61	1949	32	50	9	25–26	P/OM
**Motility/Flagella**								
Putative flagellar hook-length control protein FliK	KM596626	71	1359	16	31	9	-	IM
Flagellin	KM596627	40	300	6	17	9	-	E
FlgJ: peptidoglycan hydrolase FlgJ	KM596628	34	650	13	44	9	-	P
FlgF: Flagellar basal body rod protein	KM596629	27	147	3	19	9	-	P
FlgE: Flagellar hook protein	KM596630	47	1313	20	45	9	-	OM
FlgD: Flagellar basal body rod modification protein	KM596631	25	781	10	55	9	-	P
FlgC: Flagellar component of cell-proximal portion of basal-body rod	KM596632	15	656	7	72	9	-	P
FlgB: Flagellar basal body rod protein	KM596633	14	500	7	63	9	-	P
**Extracellular proteins**								
Putative extracellular protease	KM596661	38	387	7	29	9	24–25	E
Putative extracellular lipase	KM596662	90	1006	16	23	9	22–23	E/OM
Putative autotransporter adhesin/RTX toxin	KM596634	136	936	12	11	16	27–28	E/OM
**Unknown function**								
**Lipoproteins**								
Conserved outer membrane lipoprotein of unknown function	KM596635	12	474	8	56	9	24–25	P/OM
Conserved outer membrane lipoprotein of unknown function	KM596667	15	312	4	32	9	27–28	P/OM
Putative outer membrane associated lipoprotein	KM596636	51	1626	29	58	15	-	P/OM
Putative outer membrane associated lipoprotein	KM596637	16	121	2	18	9	24–25	P/OM
Conserved outer membrane lipoprotein of unknown function	KM596638	14	240	4	35	9	18–19	P/OM
Conserved outer membrane lipoprotein of unknown function	KM596663	24	104	1	8	9	22–23	P/OM
Conserved outer membrane lipoprotein of unknown function	KM596639	24	66	1	5	9	24–25	OM
**Others**								
Conserved outer membrane protein of unknown function	KM596640	25	284	3	14	9	22–23	OM
Conserved outer membrane protein of unknown function	KM596641	36	86	2	12	9	23–24	OM
Conserved outer membrane protein of unknown function	KM596642	84	352	6	11	9	29–30	OM
Conserved outer membrane protein of unknown function	KM596664	19	564	6	29	17	23–24	OM
Conserved putative inner membrane protein of unknown function	KM596643	67	504	10	16	15	23–24	IM
Conserved outer membrane protein of unknown function	KM596644	46	407	7	16	9	18–19	OM
Conserved protein of unknown function	KM596665	19	68	1	6	9	-	C
Conserved protein of unknown function	KM596666	56	417	6	19	16	21–22	OM

* score given by the MASCOT software, ** as given by the algorithm SignalP 3.0, *** algorithm used for subcellular localization: PsortB or Psort Gram negative bacteria. Lipoproteins localisation were predicted using LipoP1.0 (see details in materials and methods, bioinformatics). (OM: outer membrane, IM: inner membrane, P: periplasmic, E: extracellular, C: cytosolic, U: unknown)

98% and 70% of the proteins in the *V. aestuarianus* and *V. tapetis* secretomes, respectively, were predicted to have a signal peptide (see materials and methods for the algorithms used), indicating that they are periplasmic or outer membrane components (see Tables 1 & 2). Most of the proteins appeared to be normal components of the outer membrane and the periplasmic space, suggesting that they were released in the medium most probably as membrane vesicles, as was previously described for other bacteria [24]. In accordance with this hypothesis, 98% of the proteins in the case of *V. aestuarianus* and 78% in this of *V. tapetis* came from the FPLC fractions eluted in the column void volume, corresponding to materials larger than 600 kDa. Proteomic composition of these fractions appears to reflect mostly the native composition of the bacterial envelope, with no obvious specific enrichment. However, it is also possible that some of this material correspond to aggregates rather than vesicles.

The identified proteins were classified according to their biological functions (Tables 1 and 2) 1/Structural components, envelope biosynthesis and quality control, stress response, 2/energetic metabolism, 3/transporter components, 4/iron acquisition (except in *V. aestuarianus*), 5/catabolism, including chitin utilization, 6/motility, flagellar genes, 5/extracellular proteins, 6/unknown function.

### 4. Identification of known and potential virulence factors in the vibrio secretomes

The only extracellular virulence factor characterized to date in *V. aestuarianus* is the secreted zinc metalloprotease, Vam, which was shown to cause lethality of *C. gigas* oysters [11]. This protein was clearly identified and found to be quantitatively dominant in two active soluble fractions (16 and 17) in our study. More interestingly, we also identified a second extracellular protease in the *V. aestuarianus* secretome which we named Vpp (for Vam processing protease). Vpp is a homologue of Epp, a secreted protease which processes the secreted metalloprotease EmpA in *Vibrio anguillarum* [25], EmpA being a homologue of Vam. Hence, Vpp might be the Vam processing enzyme. Vpp is also a homologue of PrtV of *V. cholerae*. In *V. cholerae*, PrtV was found to play a role in resistance to grazing by natural predator, outside the host, rather than in pathogenicity to humans [26]. Further studies should clarify the role of Vpp in *V. aestuarianus*, especially as fraction 8 that contains Vpp was found to decrease oysters hemocyte adherence.

Up to now, no secreted virulence factors have been described in *V. tapetis.* The only virulence factor characterized to date is the inner membrane protein DjlA, which was shown to be required for cytotoxicity towards clam hemocytes [27]. In contrast to *V. aestuarianus*, no metalloprotease was found in the *V. tapetis* secretome, suggesting different virulence mechanisms between the two species. However, two serine proteases (i.e. KM596588 and KM596661) carrying a signal peptide (Table 2) have been identified in two different fractions. As secreted serine protease was already shown to be involved in the virulence of several pathogenic bacteria [28], these two proteins could also play a role in the pathogenesis of *V. tapetis*.

The secretomes of both vibrio species contained an extracellular triacylglycerol lipase (Table 1). This protein belongs to the same family as the phospholipase Pla1, a secreted virulence factor of *Aeromonas hydrophila* [29] and Cef, a toxin with cell elongation activity produced by *Vibrio hollisae*, which causes diarrhea in humans [30]. Phospholipases can act as potent membrane destructors and can manipulate host signalling pathways [31].

Another protein of interest is KM596634, identified in fraction 16 of the *V. tapetis* secretome, which contains the signatures of RTX toxins and autotransporters. Autotransporters are bacterial virulence factors that contain an N-terminal extracellular ("passenger") domain and a C-terminal β barrel ("β") domain that anchors the protein to the outer membrane. Upon autocleavage, the passenger domain is secreted. RTX (Repeat in toxins) toxins are virulence factors containing glycine- and aspartate-rich repeats binding Ca(2+) ions [32]. Such proteins were shown as virulence factors in other vibrio species [33], [34].

Finally, it should be noted that, contrary to *V. aestuarianus*, the *V. tapetis* secretome contains one receptor (GbpA) and several chitinases, underscoring the role of chitin as a carbon source in the environment. Besides, chitinases have already be shown to be bacterial virulence factors, eg in *Listeria monocytogenes* [35], and *Legionella pneumophila* [36]. Chitin is also a component of the shell organic matrix, and *V. tapetis* as a pathogen forms biofilms on the inner surface of the shell, typically at the level of the pallial line at the growing edge of the shell [37]. Hence, chitin use may be especially relevant to *V. tapetis* pathogenicity.

### 5. Proteins common to the *V. aestuarianus* and *V. tapetis* secretomes

Finally, the sequence of each secretome protein of a given species was compared *in silico* (using blastP) to the full proteome of the other species, allowing us to identify 21 common proteins. The results are presented in Table 3. The only potential virulence factor is the putative extracellular lipase (Pla1) already mentioned above. The other proteins corresponded to normal components of the envelope in gamma proteobacteria, and/or in the *Vibrio* genus.

**Table 3:** Proteins found in both the *V. aestuarianus* and *V. tapetis* secretomes (based on Blast of each secretome against the other).

V. tapetisGenbankaccession Nr	Protein name/function	V. aestuarianusGenbankaccessionNr
KM596604	Putative TRAP-type transport system	KM588612
KM596594	LptD	KM588604
KM596600	TolC	KM588615
KM596589	Putative lipoprotein LpoA, activatorof penicillin binding protein 1A	KM588605
KM596596	TorA	KM588608
KM596586	Tsp	KM588600
KM596603	AapJ	KM588611
KM596585	Periplasmic component of theTol-Pal system, YbgF-like	KM588602
KM596635	Conserved lipoprotein of unknown function	KM588621
KM596592	NlpI	KM588607
KM596582	OmpU	KM588597
KM596638	Conserved lipoprotein of unknown function	KM588619
KM596583	BamA (YaeT)	KM588599
KM596640	Conserved outer membraneprotein of unknown function	KM588626
KM596642	Putative outer membraneprotein of unknown function	KM588625
KM596650	LamB	KM588632
KM596647	Lpp	KM588630
KM596662	Putative extracellular lipase	KM588638
KM596652	Long-chain fatty acid outermembrane porin FadL	KM588616
KM596646	OmpA	KM588598
KM596641	Conserved outer membrane proteinof unknown function	KM588628

## Conclusion

Extracellular products, especially secreted proteins, enter in direct contact with the host cells, and play a major role in the virulence of pathogenic bacteria. As a consequence, secretomic approaches are of particular relevance to identify the proteins involved in the infection process, and several studies have been carried out for different pathogens in recent years [38]–[41]. It should be noted that to date, only two secretomes of vibrios have been reported in the literature, i.e. those of *V. coralliilyticus* [20] and *V. cholerae* [42].

In this paper, we characterized the extracellular proteome of *V. aestuarianus* and *V. tapetis*, two vibrio species pathogenic to mollusks, as a first step towards the identification of new potential virulence factors. Although the extracellular products from both species were shown to be involved in bacterial virulence, only one extracellular virulence factor has been characterized to date, in the case of *V. aestuarianus*, the Vam zinc metalloprotease [11].

This protein appeared as a major component of the *V. aestuarianus* secretome. However, we showed that a metalloprotease-free fraction (fraction 8) also displayed biological activity to hemocytes, thus suggesting the occurrence of other potential virulence factors in this species.

As the *V. tapetis* secretome does not contain any metalloprotease, the virulence mechanisms in this species are necessarily different from those in *V. aestuarianus*. In addition, we showed here that several chromatographic fractions of ECPs displayed biological activity towards oyster- and clam-hemocytes, for *V. aestuarianus* and *V. tapetis*, respectively, indicating that other factors are also responsible for the biological effects on hemocytes.

Overall, we could identify 44 and 87 different proteins in the active fractions of the *V. aestuarianus* and *V. tapetis* secretomes. Our data constitute the first valuable resource to further investigate the virulence factors of these two marine pathogen vibrios. Future works will aim at assessing the actual role of specific secreted proteins in the virulence.
